# Foliar particulate matter retention and toxic trace element accumulation of six roadside plant species in a subtropical city

**DOI:** 10.1038/s41598-023-39975-w

**Published:** 2023-08-08

**Authors:** Yazhen Chen, Yichen Xu, Xiaocui Liang, Wende Yan, Rui Zhang, Ying Yan, Shixin Qin

**Affiliations:** 1 Faculty of Life Science and Technology, Central South University of Forestry and Technology, Changsha, Hunan China; 2 National Engineering Laboratory for Applied Technology of Forestry and Ecology in South China, Changsha, Hunan China; 3 Key Laboratory of Urban Forest Ecology of Hunan Province, Changsha, Hunan China; 4 Lutou National Station for Scientific Observation and Research of Forest Ecosystem in Hunan Province, Yueyang, China; 5 Key Laboratory of Subtropical Forest Ecology of Hunan Province, Changsha, Hunan China

**Keywords:** Urban ecology, Environmental monitoring

## Abstract

As a major source of air pollution, particulate matter (PM) and associated toxic trace elements pose potentially serious threats to human health and environmental safety. As is known that plants can reduce air PM pollution. However, the relationship between PM of different sizes and toxic trace elements in foliar PM is still unclear. This study was performed to explore the association between PM of different sizes (PM_2.5_, PM_10_, PM_>10_) and toxic trace elements (As, Al, Cu, Zn, Cd, Fe, Pb) as well as the correlation among toxic trace elements of six roadside plant species (*Cinnamomum camphora*, *Osmanthus fragrans*, *Magnolia grandiflora*, *Podocarpus macrophyllus*, *Loropetalum chinense* var. rubrum and *Pittosporum tobira*) in Changsha, Hunan Province, China. Results showed that *P. macrophyllus* had the highest ability to retain PM, and *C. camphora* excelled in retaining PM_2.5_. The combination of *P. macrophyllus* and *C. camphora* was highly recommended to be planted in the subtropical city to effectively reduce PM. The toxic trace elements accumulated in foliar PM varied with plant species and PM size. Two-way ANOVA showed that most of the toxic trace elements were significantly influenced by plant species, PM size, and their interactions (*P* < 0.05). Additionally, linear regression and correlation analyses further demonstrated the homology of most toxic trace elements in foliar PM, i.e., confirming plants as predictors of PM sources as well as environmental monitoring. These findings contribute to urban air pollution control and landscape configuration optimization.

## Introduction

Particulate matter (PM) pollution in the atmosphere is becoming increasingly severe as urbanization and industrialization intensify, which has serious threats to human health and environmental safety, so it has attracted much attention^1–5^. PM is respirable air-suspended particulate matter, which can be classified as PM_2.5_ (less than or equal to 2.5 μm), PM_10_ (greater than 2.5 μm and less than 10 μm), and PM_>10_ (greater than or equal to 10 μm) based on particle size^6–8^.

Roadside plants have the potential to retain atmospheric particulate matter and improve air quality^9–12^. In recent years, researchers focused on the mechanisms of PM retention in plants and the factors affecting PM retention. For the plant itself, the leaf surface micromorphology influenced PM retention capacity^13,14^. The latest research found that the microclimate change caused by evapotranspiration on the surface of plant leaves had a significant effect on the retention of PM_2.5_^15^. The ability of plants to retain PM can be affected by planting patterns and three-dimensional configuration structures^16^. Equally important were the effects of meteorological factors on plant PM retention, for instance, rainfall and wind^17^.

Except for its ability to retain PM, the role of plants in environmental monitoring cannot be ignored^18,19^. Large amounts of toxic trace elements are present in atmospheric PM^20,21^, and PM containing toxic trace elements can float to other ecosystems^22^, and then be enriched in organisms, thereby jeopardizing their health^20^. Hence, the investigation of toxic trace elements in PM has significant implications for risk assessment and environmental monitoring. A recent study showed that plant leaf PM can substantially reflect the composition of toxic trace elements in the environment^23^. Nonetheless, there is still a lot of confusion about foliar PM and associated toxic trace elements. What is the composition of toxic trace elements in PM of different sizes retained by plants? Is it possible that plant species affect PM of different sizes to retain toxic trace elements? Who determines the concentration of toxic trace elements in foliar PM?

In this study, six roadside plants were investigated in Changsha, Hunan Province, a typical subtropical city in China. We measured the content of PM in different size fractions on their leaves and associated toxic trace elements. We also analyzed the association between PM of different sizes and toxic trace elements as well as the correlation among toxic trace elements, which is rarely seen in previous studies. The objectives of this study were to (1) evaluate the ability of different plants to retain particulate matter of different size fractions as well as toxic trace elements, (2) analyze driving factors of toxic trace element content in particulate matter, and (3) reveal potential associations between toxic trace elements from particulate matter. Our results can contribute to the knowledge of the ability of common roadside plants to retain particulate matter, toxic trace elements, and their potential associations in the subtropical area. Simultaneously, our study can provide a theoretical basis for the plant configuration in urban green belts and the application of plants in environmental monitoring. In addition, our work can provide innovative perspectives for the study of foliar PM.

## Results

### Foliar PM retention

**Figure 1:**
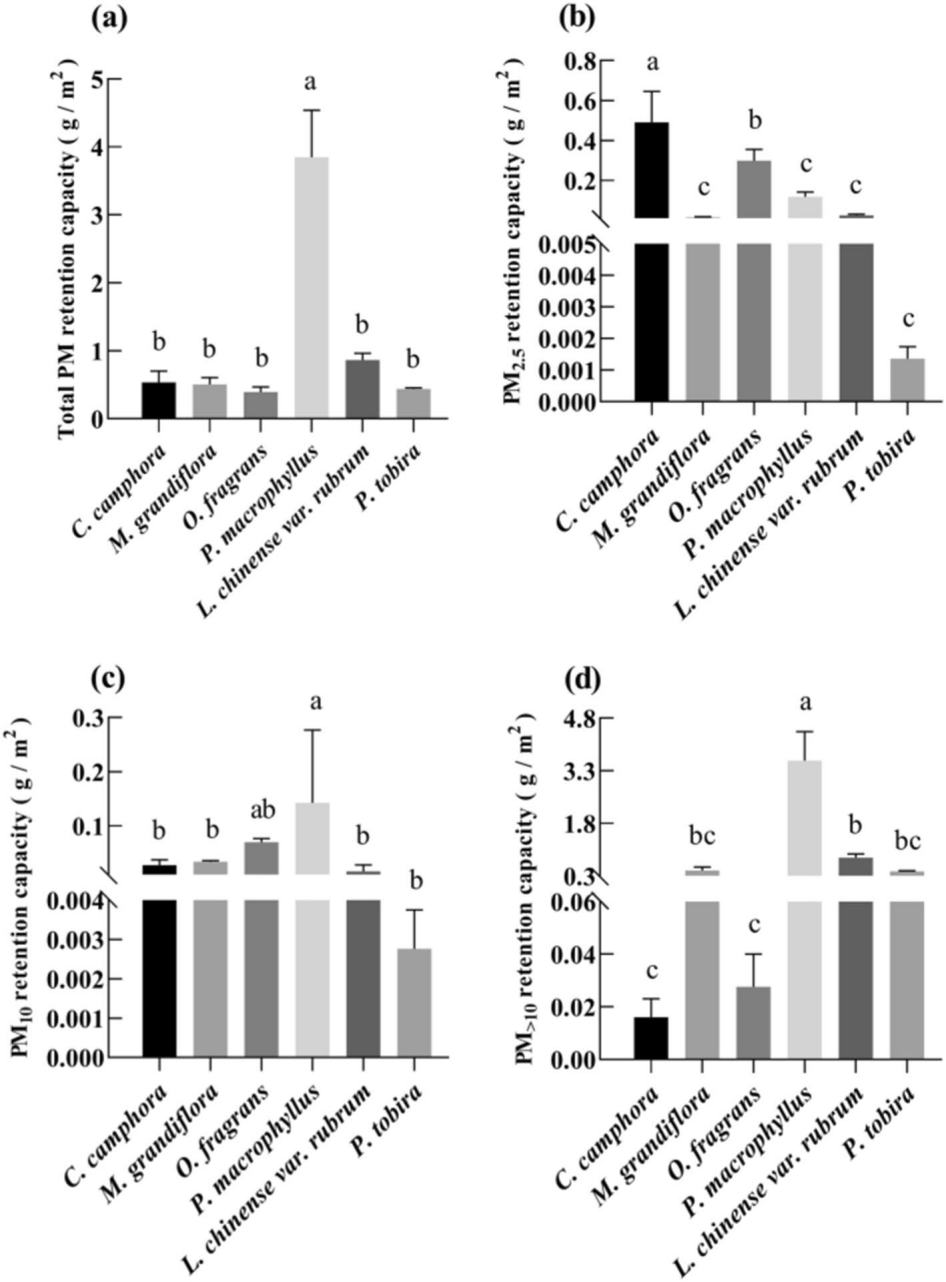
Particulate matter (PM) retention capacity of different plants. (**a**) Total PM; (**b**) PM_2.5_; (**c**) PM_10_; (**d**) PM_>10_. Different lowercase letters in the graph represent significant differences in the PM retention ability of tree species (*P* < 0.05).

Figure 1 shows the differences among plant species in the retention of PM in different size fractions. The total PM retention capacity of *P. macrophyllus* was the highest (3.8464 g/m^2^), which was significantly higher than that of the other five plants (*P* < 0.05). *Podocarpus macrophyllus* also had the greatest retention of PM_10_ (0.1426 g/m^2^) and PM_>10_ (3.5865 g/m^2^). PM_2.5_ accumulated on the leaf surface of *C. camphora* was the highest (0.4907 g/m^2^), which accounted for 91.85% of the total PM retention of *C. camphora*, while *C. camphora* had the lowest PM_>10_ retention (0.0160 g/m^2^), and it indicated that *C. camphora* was much more effective in accumulating fine particulate matter. Although *O. fragrans* had the lowest retention of total PM (0.3958 g/m^2^), PM_2.5_ retention of *O. fragrans* (0.2986 g/m^2^) was only lower than that of *C. camphora*, and significantly higher than that of *P. macrophyllus* (0.1174 g/m^2^), *L. chinense* var. rubrum (0.0254 g/m^2^), *M. grandiflora* (0.0101 g/m^2^) and *P. tobira* (0.0004 g/m^2^)*.* PM_2.5_, PM_10,_ and PM_>10_ on the leaf surface accounted for 75.43%, 17.60%, and 6.97% of the total PM retention of *O. fragrans*, which indicated that *O. fragrans* also was more effective in accumulating fine particulate matter.

### Toxic trace elements in foliar PM

**Figure 2:**
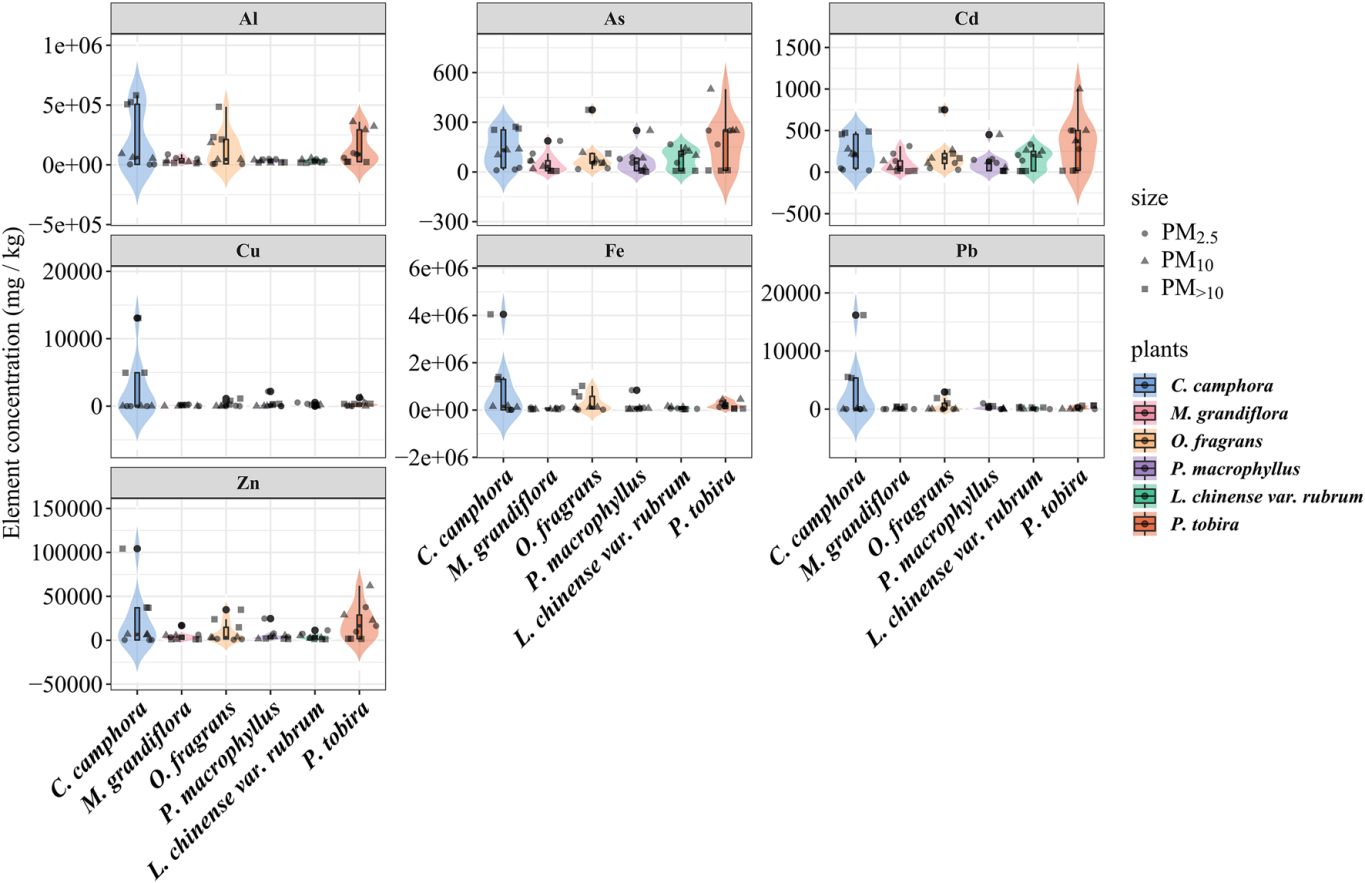
Toxic trace element concentration in foliar particulate matter of different plants. Different colors represent different plants and different shapes represent particulate matter of different sizes.

Figure 2 shows the toxic trace element concentrations in foliar particulate matter of different plants. The distribution of toxic trace elements in PM of the same particle size was different among the six roadside plants. *Cinnamomum camphora* carried the most amount of Al, Cu, Zn, Fe, and Pb in foliar PM_>10_ among the six roadside plants, and there were significant differences in the Al, Cu, Zn, Fe, and Pb concentrations in foliar PM_>10_ between *C. camphora* and the other five plant species (*P* < 0.05). The highest Al, Zn, Cd, Fe, and As concentrations were found in foliar PM_10_ of *P. tobira*, which was significantly higher than the other five plant species (*P* < 0.05). For Al, Zn, Cd, and As, *P. tobira* had the highest concentration in foliar PM_2.5_ among the six roadside plants. The highest Fe concentration in foliar PM_2.5_ was observed in *P. macrophyllus*, which was significantly higher than *C. camphora* (*P* < 0.05).

The distribution of the same element varied with plant species and particle size. For Al, *C. camphora* [PM_>10_ (537,825 mg/kg) > PM_10_ (69,319 mg/kg) > PM_2.5_ (3506 mg/kg)] and *O. fragrans* [PM_>10_ (309,648 mg/kg) > PM_10_ (91,191 mg/kg) > PM_2.5_ (7293 mg/kg)] had the highest content of Al in PM_>10_; *P. tobira* [PM_10_ (324,958 mg/kg) > PM_2.5_ (81,472 mg/kg) > PM_>10_ (25,202 mg/kg)] had the highest content of Al in PM_10_; *M. grandiflora* [PM_2.5_ (64,998 mg/kg) > PM_10_ (25,893 mg/kg) > PM_>10_ (15,055 mg/kg)] and *P. macrophyllus* [PM_2.5_ (46,844 mg/kg) > PM_10_ (28,992 mg/kg) > PM_>10_ (20,169 mg/kg)] had the highest content of Al in PM_2.5_; and there were significant differences among the three particle sizes in the same plant species (*P* < 0.05), while the Al content of *L. chinense* var. rubrum had no significant difference among the three particle sizes (*P* > 0.05). The distribution of Cd and As in the particulate matter of different plant leaves showed almost complete consistency, as evidenced by the highest levels of PM_>10_ in *C. camphora* foliage, PM_2.5_ in *M. grandiflora* foliage, PM_10_ in *P. tobira* and *L. chinense* var. rubrum foliage, and the Cd and As concentrations of *O. fragrans* and *P. macrophyllus* showed no significant difference among the three particle sizes (*P* > 0.05) (Table S1).

The results of two-way ANOVA (Table 1) showed that most of the toxic trace elements were significantly influenced by plant species, PM size, and their interactions. Al (*F* = 36.19, *P* < 0.001), Cd (*F* = 6.64, *P* < 0.001), and As (*F* = 7.63, *P* < 0.001) were more driven by the interaction of plant species and PM size, while some elements were also significantly influenced by these factors but were dominated by PM size, such as Cu (*F* = 8.70, *P* < 0.001), Pb (*F* = 11.63, *P* < 0.001), and Fe (*F* = 6.53, *P* < 0.01). More interestingly, there was no significant effect of PM size on Zn (*P* > 0.05). There was a highly significant effect of plant species on Zn in the particulate matter (*P* < 0.01).

**Table 1:** Two-way ANOVA of toxic trace elements influenced by plant species, PM size, and their interactions.

Factor	Al	Cu	Zn	Cd	Pb	Fe	As
F_P_	25.67***	5.97***	4.32**	4.59**	5.14**	4.62**	5.24**
F_S_	30.60***	8.70***	1.57	3.29*	11.63***	6.53**	3.77*
F_P_ × F_S_	36.19***	7.06***	6.73***	6.64***	5.42***	5.89***	7.63***

*P* values *< 0.05, **< 0.01, ***< 0.001, *F*_*p*_: plant species, *F*_*s*_: PM size, *F*_*P*_* × F*_*S*_: the interaction of plant species and PM size.

### Correlation analysis of toxic trace elements

**Figure 3:**
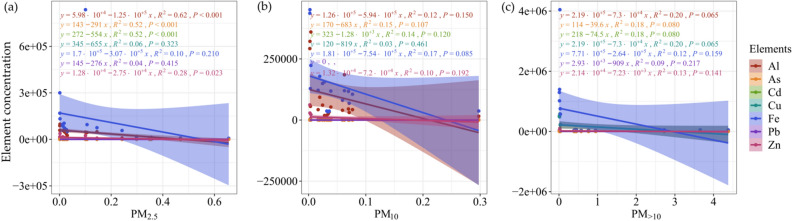
Regression analysis of toxic trace element concentrations with foliar PM retention. Different colors are used in the figure to distinguish the different toxic trace elements. Significance levels are indicated by *P* < 0.05, *P* < 0.01, and *P* < 0.001.

Linear regression analysis of toxic trace element concentrations in foliar PM and the ability of foliar PM retention showed that there was a significant correlation between Al, As, Cd, and Zn in PM_2.5_ and foliar PM_2.5_ retention (*P* < 0.05). However, there was no significant correlation between the seven elements and foliar PM_10_, PM_>10_ retention (Fig. 3).

**Figure 4:**
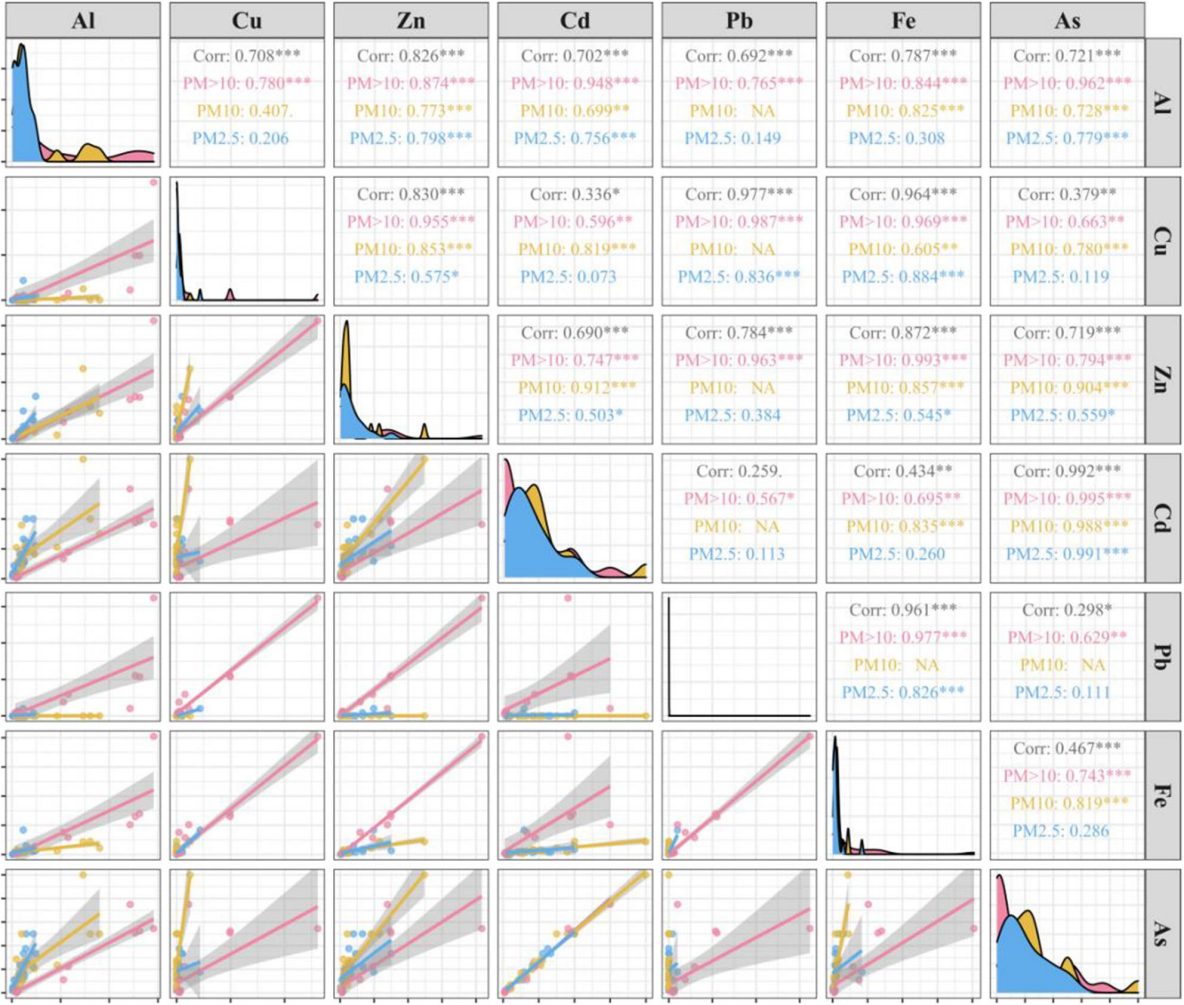
Correlation analysis between toxic trace elements. Different colors represent different sizes of particulate matter. Significance levels are indicated by *P < 0.05, **P < 0.01 and ***P < 0.001.

Most of the toxic trace elements in the particulate matter showed a highly significant correlation (*P* < 0.05), except Pb and Cd (*R* = 0.259, *P* > 0.05). However, the correlation between toxic trace elements was affected by particle size. Al and Cu were significantly correlated only at PM_>10_ (*R* = 0.780, *P* < 0.001), similarly for Al and Pb (*R* = 0.765, *P* < 0.001), Pb and Zn (*R* = 0.963, *P* < 0.001), Pb and Cd (*R* = 0.567, *P* < 0.05), and Pb and As (*R* = 0.629, *P* < 0.01). Fe and Al were not significantly correlated only at foliar PM_2.5_ (*R* = 0.308, *P* > 0.05), analogously for Cd and Cu (*R* = 0.073, *P* > 0.05), As and Cu (*R* = 0.119, *P* > 0.05), Fe and Cd (*R* = 0.260, *P* > 0.05), and Fe and As (*R* = 0.286, *P* > 0.05) (Fig. 4).

## Discussion

### Particulate matter retention among plant species

Urbanization has accelerated and increased the pressure on transportation, which has also led to a rapid escalation of particulate matter from roads^24–26^. Numerous studies have demonstrated that the roadside plant species can effectively adsorb and remove particulate matter of different sizes, which to a certain extent alleviates the environmental pollution caused by urban transportation^27–29^. In our study, *P. macrophyllus* had the highest retention of total PM, especially much more effective in accumulating large PM particles, which was consistent with previous research^30–32^. It has been proved that coniferous species can retain particulate matter more effectively than broadleaf species due to their unique leaf structure and microscopic characteristics. The microstructure of coniferous species is neat "ridged" stripes and well-arranged stomatal strips, with a large number of wrinkles and fluffy structures in the stomatal area, and the size of the fluff is nanoscale, which increases the surface area and surface roughness and is more conducive to the deposition of intercepted particles^30^. Narrow needles may be more easily hit by particles in the air than large flat leaves, compared to flattened leaves, narrow conifer needles have a much larger Stokes number and therefore have higher capture efficiency^31^. *Podocarpus macrophyllus* was not considered outstanding in its ability to retain PM_2.5_ as there were physiological differences between different conifer species^32^, and an appropriate selection of conifer species can lead to better air quality improvement^12^. Besides, we found *C. camphora* was preferable to *P. macrophyllus* and other plants in terms of their ability to retain PM_2.5_. Current studies on foliar PM_2.5_ capture capacity for evergreen broad-leaved trees (*C. camphora*) are inconclusive. Some researchers found that *C. camphora* did not effectively capture small-size particulate matter^33,34^. But others proved that evergreen broad-leaved trees had greater adsorption capacity of PM_2.5_ than coniferous trees^31^, and this is consistent with our results. The reason was that the leaf stomata can adsorb PM_2.5_^31^, and the broad-leaved trees (*C. camphora*) have more stomatal density than coniferous trees (*P. macrophyllus*)^35–37^, so *C. camphora* showed very high adsorption capacity of PM_2.5_. In addition, the subtle changes in ambient water vapor brought by the evapotranspiration of plant leaves would also be effective in retaining PM_2.5_^15^. In our study, *P. macrophyllus* had the highest ability to retain PM, and *C. camphora* excelled in retaining PM_2.5_. Therefore, our results suggest that the combination of *P. macrophyllus* and *C. camphora* was highly recommended to be planted in the subtropical city to effectively reduce PM from the air.

### Toxic trace elements in foliar particulate matter

Previous studies mainly focused on the adsorption of particulate matter by plants and the factors influencing the reduction of PM^31^, but the relationship between PM of different sizes and toxic trace elements in foliar PM remains unclear. Our results demonstrated that there was a "particle size effect" for most toxic trace elements in foliar PM. All of the trace element was influenced by particle size except Zn. However, the mechanism of the effects of particle size on trace elements is still unclear, which can be a direction of future research. Additionally, the different content of the same elements in particulate matter of the same particle size indicates that the particulate matter may be from different sources, such as the elemental composition of particulate matter originating from industrial areas and non-industrial areas and their content was completely different^34^. To avoid the influence of environmental factors, all the trees studied were in the same road adjacent area. Therefore, the most likely reason is that different plant leaves absorb toxic trace elements from the same source of particulate matter, leading to variations in their concentrations. There was further evidence from the two-way ANOVA results that plant species and the interaction of plant species and particle size had a highly significant effect on the seven toxic trace elements, which has rarely been proved by such methods in previous studies^38,39^. Different plants generally have different foliar microstructures, i.e., some plants are more likely to absorb trace elements from large particulate matter on the leaf surface^40,41^, while others are more inclined to fine particulate matter, such as PM_2.5_^42^. All toxic trace elements in PM_>10_ on *C. camphora* foliage in our study were higher than those in other particle-size particulate matter. Some plants are selective in the adsorption of particulate matter due to their leaf physicochemical factors, thus explaining why *C. camphora* has the worst PM_>10_ adsorption capacity but its ability to retain toxic trace elements is better^43^.

### Association between toxic trace elements and their association with foliar retention capacity of particulate matter

In theory, the more particulate matter retained on the leaf surface, the more toxic trace elements should be accumulated accordingly^39,44^. However, the linear regression results showed that some toxic trace elements showed a significant negative correlation with the particulate matter content when the particle size was less than 2.5 µm. This result indicated the toxic trace element may be absorbed by plant leaves. Furthermore, it has been proved that plant species can affect the content of toxic trace elements in foliar particulate matter through their microstructures as well as particulate matter selection^33^. There is a finding that plant health also affects their ability to retain particulate matter. At the same time, particles can also affect plant growth, photosynthesis, respiration, and transpiration when the particles on the leaf surface reach a certain amount^45^. Therefore, the differences in foliar PM retention capacity and toxic trace element accumulation in PM can also be attributed to uncertainties associated physical and chemical processes involved in plant–particles interactions.

In our study, the phenomenon of a closely association between toxic trace elements in leaf PM was comparable to the results of most studies^46–49^. Even though the plant leaf samples were collected in the same time–space, there were some discrepancies in the sources of PM even in the same area, for instance, roadside PM may originate from car exhaust emissions or road PM^36^. There are records suggesting that Cd, Cu, Pb, and Zn generally originate from road PM^50,51^, while vehicle exhaust emissions bring As, Cd, and Pb^52,53^. The highly significant correlation between As and Cd in all particle sizes in this study further confirms the above statement and also illustrates that PM retention on plant foliage can reflect fairly well the sources as well as the distribution of toxic trace elements in the environment of roadsides^54,55^.

## Conclusions

In this study, *P. macrophyllus* had the strongest ability to retain PM, and *C. camphora* excelled in retaining PM_2.5_. The combination of *P. macrophyllus* and *C. camphora* is highly recommended to be planted in subtropical cities to effectively reduce PM. PM size and plant species and their interactions played an important role in the toxic trace element concentration of foliar particulate matter. Additionally, the toxic trace element concentration of foliar particulate matter is co-dependent on the physiological characteristics of the plant itself as well as environmental sources. The plants can predict the source of PM by toxic trace elements. This study provides a new reference for city greening and air pollution control.

## Materials and methods

### Study area

Changsha is located in the eastern part of Hunan Province (111° 53′–114° 15′ E, 27° 51′–28° 40′ N), which is in the low latitude zone with a subtropical monsoon climate. The annual average temperature is 17.2 °C, and the annual precipitation is 1361.6 mm in the city. The annual average PM_2.5_ concentration in 2017 was higher than the limit value of the Chinese Secondary Standard of Environmental Air Quality Standards (GB 3095-2012) (35 μg/m^3^), and the annual average PM_10_ concentration is equal to the limit value of the Chinese Secondary Standard of Environmental Air Quality Standards (GB 3095-2012) (70 μg/m^3^).

### Sample collection

**Figure 5:**
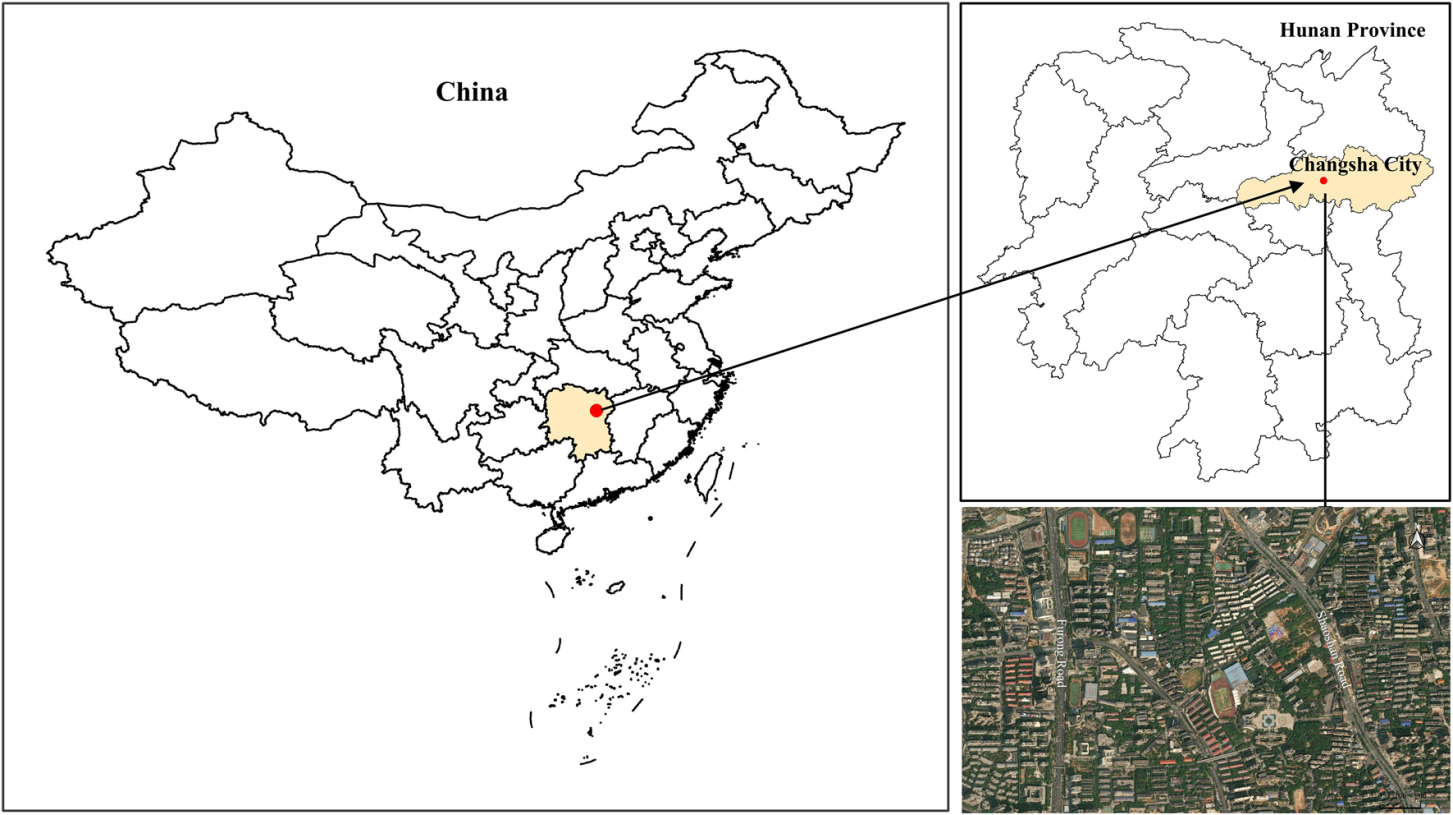
Location of the research areas. ^†^Software&Version：QGIS 3.32.0. URL:https://server.arcgisonline.com/arcgis/rest/services/World_Imagery/MapServer/tile/{z}/{y}/{x}.

By surveying greening tree species in Changsha, we selected six typical evergreen broad-leaved and coniferous greening tree species in Changsha to monitor and analyze their PM retention capacity, including four arbor species: *Cinnamomum camphora*, *Osmanthus fragrans*, *Magnolia grandiflora* and *Podocarpus macrophyllus*, and two shrub species: *Loropetalum chinense* var. rubrum and *Pittosporum tobira* (Table 2). The plants we selected were not listed as national and provincial key protected wild plants in China nor threatened species on the IUCN Red List. Therefore, no specific permissions or licenses were needed for the sampling of plants for research purposes according to the regulations of the People’s Republic of China on the protection of wild plants. During the sampling process, we followed the local sampling guideline to ensure no substantial harm to the collecting individual. The plant leaf samples were collected on both sides of the main roads (Shaoshan Road and Furong Road) in Changsha City (Fig. 5) on December 25, 2017, when there was no previous rainfall for more than 7 days. The width of the green belt was about 3 m, and the plants in the green belt included arbors, shrubs, and herbs with consistent habitat conditions. To avoid the impacts caused by the differences in location and distance, the samples were selected within 3 m from the road and all samples were collected within 1 day. The height of sampling was 2–6 m for arbors and 0.5–3 m for shrubs. For each species, ten trees were selected as samples, and the leaves were collected randomly in four different directions (east, west, south, and north) at the upper, middle, and lower positions of the tree canopy. The leaves collected are required to be healthy and free of pests and diseases. After sample collection, the samples were carefully stored in sealed plastic bags and immediately transferred to a refrigerator at 4 °C for subsequent experiments and analysis. Meanwhile, the diameter at breast height (DBH) and basal diameter (BD) of plants were measured with a diameter at breast height ruler, the height of each tree was measured with a height measuring device, and the width of the crowns was measured with a ruler in two directions: north–south and east–west.

**Table 2:** Basic information of the investigated tree species.

Items	No	Species	Family	DBH (BD) /cm	Crown diameter/m	Height/m	Leaf surface features
Evergreen broad-leaved trees	1	*Cinnamomum camphora*	Lauraceae	27.65 ± 10.84	7.51 ± 2.02	7.85 ± 2.37	Glabrous on both surfaces or slightly microvillus below when young
	2	*Magnolia grandiflora*	Magnoliaceae	12.80 ± 2.17	4.66 ± 0.65	5.87 ± 0.98	Lower surface densely covered with brown or gray-brown villus
	3	*Osmanthus fragrans*	Oleaceae	21.33 ± 2.00	5.51 ± 0.68	5.70 ± 0.81	Glabrous on both sides, concave above midrib, and convex below midrib
Evergreen coniferous trees	4	*Podocarpus macrophyllus*	Podocarpaceae	8.07 ± 2.37	2.05 ± 0.56	3.23 ± 0.25	Midrib raised
Evergreen broad-leaved shrubs	5	*Loropetalum chinense* var. rubrum	Hamamelidaceae	10.80 ± 1.98	3.46 ± 0.43	0.90 ± 0.24	Stellate hairs on both sides
	6	*Pittosporum tobira*	Pittosporaceae	–	–	1.60 ± 0.18	Villus on both sides of the tender leaf, and then it becomes bald

^†^Data are means ± standard deviation (SD).

### Extraction and processing of foliar particulate matter

The retention of particulate matter on the surface of plant leaves was determined by using the microporous membrane weighing method^53^. The number of test leaves was determined according to leaf size and type to ensure that the experimental leaf samples were adequate and randomized, and three replicates of each plant were required. In general, the larger leaves in broadleaf needed 30–50 pieces, and the smaller leaves needed 150–300 pieces. Put the selected plant leaf samples into a beaker with deionized water for half an hour, and then carefully clean all the PM on the leaves with a small brush. Next, pinch out the leaves with pointed tweezers (be careful not to damage the leaves) and rinse them three times with an appropriate amount of deionized water, then put them on newspaper to dry. Finally, the dried leaves were scanned with a scanner (Epson scanner 11000G) to obtain the leaf projection, and export all the leaf scanned images, then opened Image J software, the edges of the leaves were circled and the leaf area was calculated by the software. To reduce the error, the area of each leaf needed to be calculated three times and get the average value.

The solution was filtered through the dried and weighed microporous membrane of 10 μm pore size, and then the filtrate was filtered through the dried and weighed microporous membrane of 2.5 μm and 0.1 μm pore size to obtain three different particle size levels of PM_>10_, PM_10_ and PM_2.5_ using the same procedure as above. Before and after each filtration, the microporous filter membrane was put in an oven at 60 °C^31^ and dried to a constant mass (two measurements ≤ 0.0002 g), and weighed on a balance with an accuracy of one ten-thousandth.

The plant leaf PM retention was determined by the mass difference method. The calculation method was as follows:

PM retention per unit leaf area (g/m^2^):

1 $${\text{M}} = \left( {{\text{M}}_{{2}} - {\text{M}}_{{1}} } \right)/{\text{S}}$$

In the formula, M is the retention of particulate matter per unit leaf area (g/m^2^); M_1_ is the dried mass of filter membrane before filtration (g); M_2_ is the dried mass of filter membrane and particulate matter after filtration (g); S is the total area of test leaf samples (m^2^).

### Toxic trace element accumulation of foliar particulate matter

The content of toxic trace elements in PM of plant leaf surface was determined by aqueous solubilization digestion. Approximately 0.2 g of filter membranes were digested in a glass tube with HNO_3_ and HCl mixture (1:3 v/v ratio) using a graphite heater furnace (Polytech PT60, Polytech 3 Instrument, Beijing, China) and kept the mixture in a slightly boiling state for 2 h. Then the content of toxic trace elements in the digestion solution was determined by the Inductively coupled plasma optical emission spectrometer (ICP-OES, Optima 8300, USA), and the detection limit of this instrument is 0.001–0.1 mg/L. The national standard GSS-5 (Hunan soil) was selected for quality control and the measured standard recoveries ranged from 85.1 to 114%. In this experiment, As, Al, Cu, Zn, Cd, Fe, and Pb, which are common toxic trace elements in PM, were measured. The standard solutions were selected from the national standard samples of single-element standard solutions: As (GSB 04-1714-2004), Al (GSB 04-1713-2004), Cu (GSB 04-1725-2004), Zn (GSB 04-1761-2004), Cd (GSB 04-1721-2004), Fe (GSB 04-1726-2004) and Pb (GSB 04-1742-2004), with the concentration of 1000 μg/ml.

### Statistical analysis

One-way ANOVA was used to compare the differences in the PM retention capacity of different plants. Two-way ANOVA was used to reveal the underlying drivers of toxic trace element concentrations in particulate matter. Linear regression was used to explore the relationship between toxic trace element concentrations of particulate matter and the ability of the foliage to retain particulate matter. Correlation analysis based on Pearson's method was used to examine the association among different toxic trace elements. GraphPad Prism 8 as well as the R-based packages "ggplot2" and "GGally" were used for data visualization. All statistical analyses in our work were done in R 4.1.1 (R-Core-Team, 2013).

### Ethical approval

All procedures were followed in compliance with institutional, national, and international rules and legislation. The plants we selected were not listed as national and provincial key protected wild plants in China nor threatened species on the IUCN Red List. Therefore, no specific permissions or licenses were needed for the sampling of plants for research purposes according to the regulations of the People’s Republic of China on the protection of wild plants.

### Supplementary Information

Supplementary Information.

## Data Availability

The datasets generated and/or analyzed during the current study are not publicly available due [The data may be used in our next analysis] but are available from the corresponding author on reasonable request.
