# Supplementary material for: Foliar particulate matter retention and toxic trace element accumulation of six roadside plant species in a subtropical city
Source: Sci Rep. 2023 Aug 8;13:12831. doi: 10.1038/s41598-023-39975-w (PMC10409817; doi:10.1038/s41598-023-39975-w)
Supplement: Supplementary file 1 — Supplementary Information. [file 41598_2023_39975_MOESM1_ESM.docx]

Supplementary Materials for

**Foliar particulate matter retention and toxic trace element accumulation of six roadside plant species in a subtropical city**

Yazhen Chen^1,2^, Yichen Xu^1,2^, Xiaocui Liang^1,2,3,4,5*^, Wende Yan^1,2,3,4,5*^, Rui Zhang^1^, Ying Yan^1^, Shixin Qin^1^

1 Faculty of Life Science and Technology, Central South University of Forestry & Technology, Changsha, Hunan, China

2 National Engineering Laboratory for Applied Technology of Forestry & Ecology in South China, Changsha, Hunan, China

3 Key Laboratory of Urban Forest Ecology of Hunan Province, Changsha, Hunan, China

4 Lutou National Station for Scientific Observation and Research of Forest Ecosystem in Hunan Province, Yueyang, China

5 Key Laboratory of Subtropical Forest Ecology of Hunan Province, Changsha, Hunan, China

*** Correspondence:**

Xiaocui Liang, Wende Yan

[t20081388@csuft.edu.cn,](mailto:xcliang1026@126.com,) t20001421@csuft.edu.cn

**Contents**

Table S1. The toxic trace element concentrations at different sizes of particulate matter (PM) of six plant species. Data are means ± standard deviation (SD), n=3. Diﬀerent lowercase letters indicate signiﬁcant diﬀerences among plant species at the *P* ≤ 0.05 level, and diﬀerent capital letters indicate signiﬁcant diﬀerences among PM sizes at the *P* ≤ 0.05 level.

| **Plants** | **Size** | **Al** | **Cu** | **Zn** | **Cd** | **Pb** | **Fe** | **As** |
| --- | --- | --- | --- | --- | --- | --- | --- | --- |
|  |  |  |  |  |  |  |  |  |
| ***Cinnamomum camphora*** | **PM_2.5_** | 3506 ± 678 dC | 0 ± 0 aB | 394 ± 14 bB | 32 ± 13 cC | 0 ± 0 aB | 7860 ± 1134 bB | 17 ± 7 cC |
|  | **PM10** | 69319 ± 21219 bB | 0 ± 0 aB | 6521 ± 417 bB | 233 ± 38 bB | 0 ± 0 aB | 149172 ± 30118 bB | 124 ± 19 bB |
|  | **PM_>_10** | 537825 ± 39496 aA | 7658 ± 4686 aA | 59523 ± 38736 aA | 472 ± 17 aA | 9018 ± 6204 aA | 2246275 ± 1558938 aA | 263 ± 9 aA |
| ***Magnolia grandiflora*** | **PM2.5** | 64998 ± 19570 abA | 139 ± 127 aAB | 8895 ± 6973 abA | 223 ± 90 bA | 0 ± 0 aB | 95197 ± 9691 abA | 122 ± 61 bA |
|  | **PM10** | 25893 ± 7031 bB | 0 ± 0 aB | 3922 ± 2739 bA | 80 ± 48 bB | 0 ± 0 aB | 35766 ± 1834 cB | 40 ± 25 bB |
|  | **PM_>_10** | 15055 ± 2212 cB | 153 ± 1 bA | 1201 ± 57 bA | 14 ± 2 bB | 408 ± 10 bA | 35529 ± 2758 bB | 7 ± 1 bB |
| ***Osmanthus fragrans*** | **PM2.5** | 7293 ± 1105 dB | 21 ± 36 aB | 1175 ± 448 bB | 61 ± 43 cA | 0 ± 0 aB | 29262 ± 24355 abB | 31 ± 21 cA |
|  | **PM10** | 91191 ± 81688 bB | 0 ± 0 aB | 3063 ± 526 bB | 179 ± 80 bA | 0 ± 0 aB | 114526 ± 7756 bcB | 81 ± 33 bA |
|  | **PM_>_10** | 309648 ± 152609 bA | 708 ± 455 bA | 24472 ± 9999 bA | 380 ± 322 aA | 1944 ± 973 bA | 784111 ± 222216 bA | 181 ± 171 aA |
| ***Podocarpus macrophyllus*** | **PM2.5** | 46844 ± 2342 bcA | 725 ± 1257 aA | 12118 ± 11082 abA | 139 ± 24 bcA | 333 ± 577 aA | 335804 ± 433959 aA | 74 ± 17 bcA |
|  | **PM10** | 28992 ± 12263 bB | 0 ± 0 aA | 3666 ± 1914 bA | 209 ± 212 bA | 0 ± 0 aA | 62193 ± 22523 bcA | 120 ± 117 bA |
|  | **PM_>_10** | 20169 ± 821 cB | 277 ± 71 bA | 1789 ± 268 bA | 15 ± 2 bA | 461 ± 101 bA | 51891 ± 1022 bA | 5 ± 4 bA |
| ***Loropetalum chinense* var. rubrum** | **PM2.5** | 36093 ± 11123 cA | 367 ± 152 aA | 7091 ± 4396 abA | 226 ± 99 bA | 139 ± 241 aAB | 61500 ± 11286 abB | 113 ± 56 bA |
|  | **PM10** | 39140 ± 18852 bA | 63 ± 110 aB | 3429 ± 1602 bA | 237 ± 33 bA | 0 ± 0 aB | 135809 ± 18368 bA | 123 ± 22 bA |
|  | **PM_>_10** | 18545 ± 1247 cA | 197 ± 31 bAB | 1362 ± 81 bA | 13 ± 1 bB | 293 ± 22 bA | 38072 ± 3899 bB | 7 ± 0 bB |
| ***Pittosporum tobira*** | **PM2.5** | 81472 ± 20345 aB | 194 ± 268 aA | 21280 ± 14523 aAB | 426 ± 128 aAB | 139 ± 241 aB | 200752 ± 88086 abAB | 222 ± 48 aA |
|  | **PM10** | 324958 ± 34034 aA | 417 ± 722 aA | 37833 ± 21143 aA | 625 ± 331 aA | 0 ± 0 aB | 370542 ± 127663 aB | 333 ± 144 aA |
|  | **PM_>_10** | 25202 ± 1335 cC | 338 ± 13 bA | 1681 ± 170 bB | 18 ± 2 bB | 609 ± 11 bA | 62210 ± 2875 bA | 9 ± 1 bB |
